# Similar mechanisms of temporary bindings for identity and location of objects in healthy ageing: an eye-tracking study with naturalistic scenes

**DOI:** 10.1038/s41598-022-13559-6

**Published:** 2022-07-01

**Authors:** Giorgia D’Innocenzo, Sergio Della Sala, Moreno I. Coco

**Affiliations:** 1 Centro de Investigação em Ciência Psicológica (CICPSI), Faculdade de Psicologia, Universidade de Lisboa, Lisbon, Portugal; 2 Human Cognitive Neuroscience, Department of Psychology, University of Edinburgh, Edinburgh, UK; 3 Department of Psychology, “Sapienza” University of Rome, Rome, Italy; 4 IRCCS Santa Lucia, Rome, Italy

**Keywords:** Psychology, Human behaviour, Cognitive ageing, Object vision

## Abstract

The ability to maintain visual working memory (VWM) associations about the identity and location of objects has at times been found to decrease with age. To date, however, this age-related difficulty was mostly observed in artificial visual contexts (e.g., object arrays), and so it is unclear whether it may manifest in naturalistic contexts, and in which ways. In this eye-tracking study, 26 younger and 24 healthy older adults were asked to detect changes in a critical object situated in a photographic scene (192 in total), about its *identity* (the object becomes a different object but maintains the same position), *location* (the object only changes position) or *both* (the object changes in location and identity). Aging was associated with a lower change detection performance. A change in identity was harder to detect than a location change, and performance was best when both features changed, especially in younger adults. Eye movements displayed minor differences between age groups (e.g., shorter saccades in older adults) but were similarly modulated by the type of change. Latencies to the first fixation were longer and the amplitude of incoming saccades was larger when the critical object changed in location. Once fixated, the target object was inspected for longer when it only changed in identity compared to location. Visually salient objects were fixated earlier, but saliency did not affect any other eye movement measures considered, nor did it interact with the type of change. Our findings suggest that even though aging results in lower performance, it does not selectively disrupt temporary bindings of object identity, location, or their association in VWM, and highlight the importance of using naturalistic contexts to discriminate the cognitive processes that undergo detriment from those that are instead spared by aging.

## Introduction

There is some evidence suggesting that the ability to efficiently process information in visual working memory (VWM) in support of ongoing tasks^1,2^ can decline with age^3–5^. Studies investigating VWM in young adults have often employed a change detection paradigm^6^, whereby participants are first presented with a visual context, usually an array of geometrical objects (e.g., lines), and then, after a brief interval (e.g., 900 ms), are asked whether they noticed a change in one or more low-level properties (e.g., colour, orientation) of such objects^7,8^. An established finding is that changes to single features are better remembered than combinations^9–11^. Encoding and maintaining combinations of features such as the relationship between objects and locations in VWM may be costly, especially for older adults, who often display a reduced accuracy and an increased time compared to younger adults^12–15^ (but see^4,16–20^ for evidence of preserved abilities in this task).

Age-related deficits in VWM may relate to maintaining the association between the spatial *location* of an object and its *identity*, rather than just bindings between nonspatial features^16,20,21^. Object identity and location are processed by two different neural pathways—the dorsal and the ventral stream, also known as the “where” and the “what” pathways, respectively^22,23^. Consistent with this division, observers need to encode the identity and location of objects to be able to bind this information together^24–26^. These processes rely at least in part on the hippocampus^27–29^, which suffers from decreased activation due to aging^30^. Consequently, temporary memory about the identity and location of visual objects has been investigated throughout the lifespan, to determine whether and how aging may alter these mechanisms^4,31,32^.

Some studies have shown that older adults remember the locations of objects comparably to younger adults^33,34^ while others argued that memory for object locations is affected by aging, whereas memory for object identity is mostly spared^12,25^. Other research has shown that older adults display impaired memory for both types of information, especially when remembering object–location associations^35–37^. Mitchell and colleagues^30^, for example, found that older adults were significantly impaired when required to remember object–location information concurrently, but they performed similarly to younger adults when tested on location or identity information in isolation. An opposite pattern was reported by Pertzov and colleagues^38^, who found degraded memory for both identity and location information with age, but no evidence of degraded object–location binding.

There currently is no clear consensus as to whether the reductions in short-term memory that typically accompany healthy aging^39,40^ selectively impair memory for the identity or the location of visual objects, or the ability to bind this information together. These discrepancies may arise because VWM abilities have mainly been studied using arrays of simple geometrical shapes^13,14,16^, abstract objects^38^, drawings of real objects arranged within grids^12,34–37,41^, or computer-generated images of simple backgrounds^25^. Although artificial stimuli guarantee a tight control over a simple set of parametrizable low-level visual features (e.g., shape, color, size), they lack a structured context, which can mediate the processing of objects and locations^21,42–45^. Complex scenes (e.g., photographs), for example, comprise several objects (e.g., a knife, a table, etc.) structured into a coherent context (e.g., a kitchen scene), which can facilitate object recognition (e.g., see^46^ for a review on the importance of scenes for cognition, and^47^ for recent neurophysiological evidence). Therefore, such rich naturalistic contexts (e.g., photographs) may better support the encoding of object information in short-term memory and thus reduce the likelihood to encounter impaired abilities.

The few studies investigating this topic and using a change detection task situated in naturalistic contexts seem inconclusive. Rizzo and colleagues^48^ asked participants to detect changes to photographs of roads taken from a driver’s perspective and reported impaired performance in older adults. Costello and colleagues^49^ presented participants with photographs of natural scenes; they found that older adults were slower and less accurate in detecting changes to objects in the scene than younger adults, but these differences were reduced when the general cognitive slowing naturally associated with aging^50–52^ was considered. Thus, the primary goal of the present study is to investigate the ability of younger and older adults to successfully form temporary bindings about the identity and location of objects situated in naturalistic scenes. We aim to provide novel evidence about short-term visual memory mechanisms for object identities, locations, and their associations in naturalistic contexts.

When exploring the impact of age on encoding and maintaining object information in VWM, gaze behaviour provides additional insights compared to manual responses^53,54^. Eye movements can reveal links between the allocation of overt attention and memory processes^55,56^, and thus help explain differences associated with cognitive aging, e.g.^57^, also in VWM, as assessed, for example, using change detection tasks^15,58^. Thus, the second aim of the present study is to investigate how overt attention supports the successful detection of changes in the identity and location of objects situated in naturalistic scenes and whether it is revelatory of age-related differences.

We compared the ability of healthy older and younger adults to detect changes in naturalistic (photographic) scenes occurring to a single object feature (i.e., location *or* identity) or a combination of features (i.e., location *and* identity) while we monitored their eye movement behaviour (see “Methods” for details). The critical object was placed to the left or the right of the scene, i.e., in extra-foveal vision from the screen centre. This was done to make sure that participants purposedly selected the critical object as the saccade target and to avoid any asymmetry in memory performance between central and peripheral locations^59^. If recalling object–location associations is costly, as observed in artificial displays, we would expect performance to be lowest when both the identity and the location of the critical object are changed also in naturalistic scenes, especially in older adults, e.g.^13^. However, naturalistic scenes provide contextual and relational information about the objects therein, which may facilitate memory for the seen items^45,60,61^. Therefore, if the maintenance of VWM representations is facilitated in naturalistic scenes, we may observe an opposite effect, whereby detection accuracy should be highest when both identity and location of the critical object change, especially in younger adults, who may remember the identity of the objects better than older adults^62^.

In terms of eye-movement behaviour, change detection can be framed as a search task^63,64^, where observers inspect the scenes intending to remember the identities and locations of the objects therein^65^. We considered three eye movement measures that can be taken as indexes of a “search” strategy to detect the change (see “Methods” for details) and expected them to be differently modulated by the type of change implemented. Specifically, larger incoming saccades should be observed when objects change spatial location compared to when only the identity is changed—especially in younger adults, who have a more efficient perceptual span^15,66^. Moreover, when the object changes in location and identity, we expect longer latencies of the first fixation compared to when it only changes in identity, as searching for a conjunction of features is known to take longer than for a single feature, especially in older adults^67^. Finally, for changes involving relocation of the object we expect memory-based effects on the orienting of visual attention^55,68^. Participants should be faster and require less inspection time when they remember where the object was during the study phase, compared to when they further explore the scene to detect where the object now is.

In investigating eye-movement behaviour, we also evaluated the influence of low-level visual saliency, which is known to interact with high-level processes and modulate the allocation of overt attention (^69–71^; and see^72^ for evidence of faster search times of visually salient targets in photographic scenes). However, it is also known that this effect highly depends on the demands of the task, with overt attention being more strongly guided by low-level visual saliency in weakly structured tasks, e.g., free viewing^73,74^. Thus, as cognitive factors and the contents of VWM can override the effects of saliency in directing attention^74–77^, we do not expect visual saliency to result in any systematic relation between the type of change (e.g., identity or location) being correctly recalled and the eye-movement responses that are associated with it.

## Methods

### Participants

Twenty-six young adults (9 men) between the age of 18 and 33 (mean age 24.9 years), and twenty-four older adults (11 men) between the age of 67 and 86 (mean age 72.7 years) took part in the experiment after providing written informed consent and received an honorarium of £7 per hour. The data for an additional older participant was collected but discarded from further analyses because their performance on the task was at chance under a binomial test. All participants had normal or corrected-to-normal vision, and none reported a history of neurological disorders. Participants were assessed on a battery of neuropsychological tests tapping into different cognitive functions from verbal memory (e.g., Rey Auditory Verbal Learning Test) to visual object perception (Visual Object and Space Perception Battery); see Table 1 for the comparison of older and younger participants’ performance on these tests. Ethical approval for the study was obtained by the Ethics Committee of the Department of Psychology at the University of Edinburgh before starting the data collection. The study was performed in line with the principles of the Declaration of Helsinki.

**﻿Table 1:** Comparison of younger and older adults on a battery of neuropsychological tests spanning general cognition (MMSE), executive control (TMT), retrieval fluency (BNT, COWA), verbal working memory (RAVLT), and visuospatial abilities (VOSP).

Neuropsychological test		Older	Younger	p-value
Mini Mental State Examination		29.16 (0.74)	29.46 (0.58)	0.27
Boston Naming Test	Raw score	25.72 (3.31)	26.85 (4.23)	0.31
Trail Making Test	A	27.23 (6.07)	20.38 (3.72)	0.0001
	B	84.92 (54.1)	41.42 (12.34)	0.0001
Controlled Oral Word Association	Letters	56.25 (9.16)	49.5 (10.81)	0.02
	Animals	23.33 (4.96)	26.38 (4.16)	0.02
	Total	79.04 (12.4)	75.85 (12.6)	0.37
Ray Auditory Verbal Learning Test	Total	50.42 (8.03)	54.46 (8.34)	0.09
	Forgotten	− 2.29 (1.49)	− 1.12 (1.99)	0.02
	Recalled	21 (4.16)	23.69 (2.77)	0.01
	Rejected	15.21 (4.72)	14.96 (3.49)	0.84
Visual Object Space Perception	Incomplete Letters	19.17 (0.76)	19.54 (0.58)	0.06
	Silhouettes	20.08 (4.46)	21.38 (4.23)	0.3
	Object Recognition	18 (1.32)	17 (1.83)	0.03
	Object Progressive	8.38 (3.21)	9.77 (3.01)	0.12
	Dot Position	19.75 (0.68)	19.81 (0.4)	0.72
	Number Position	9.21 (1.64)	9.54 (0.95)	0.39

### Design

**Figure 1:**
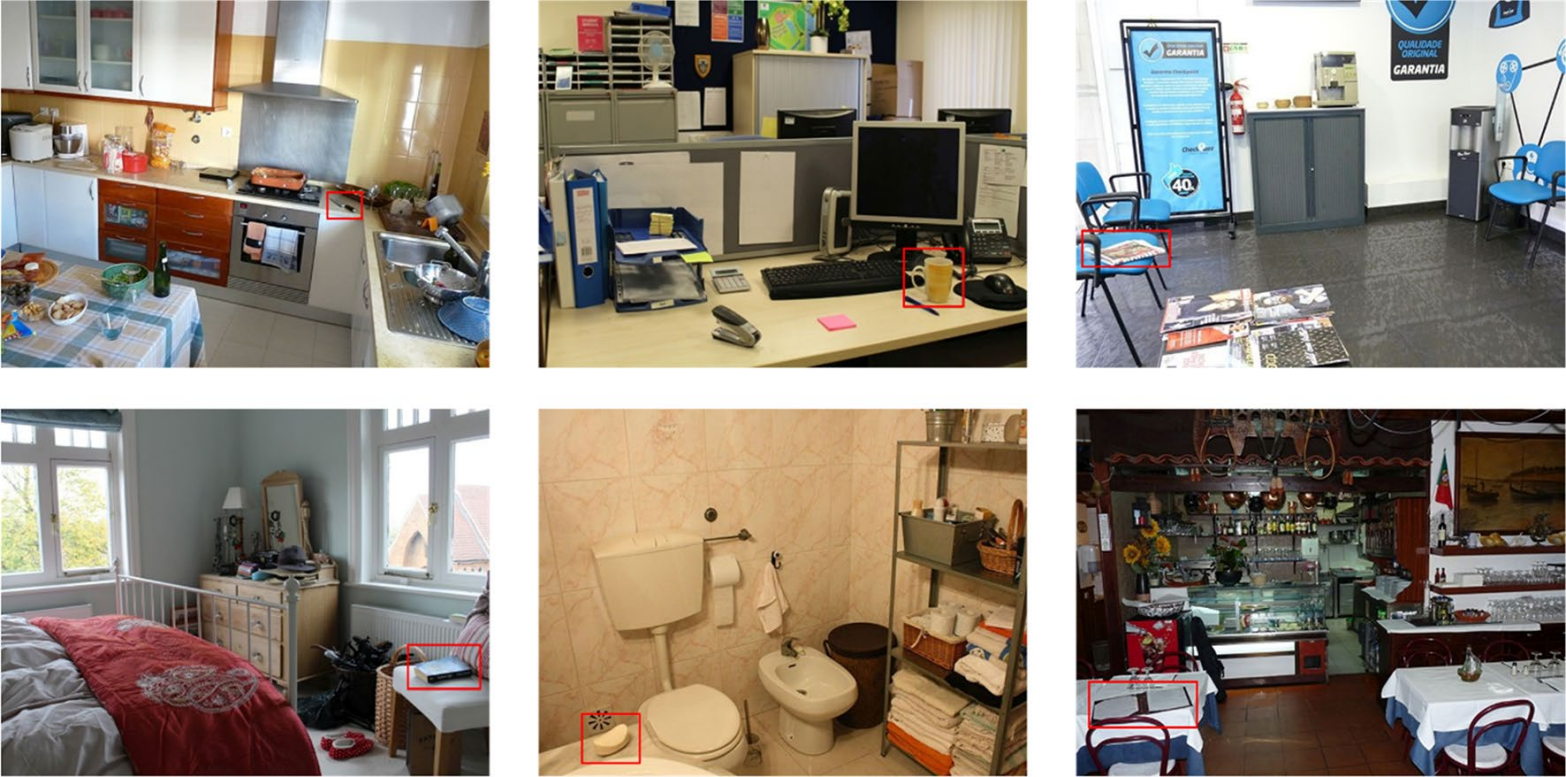
Examples of the type of scenes used in the study. For illustration, critical objects are shown surrounded by a red square, which was not present during the experiment.

**Figure 2:**
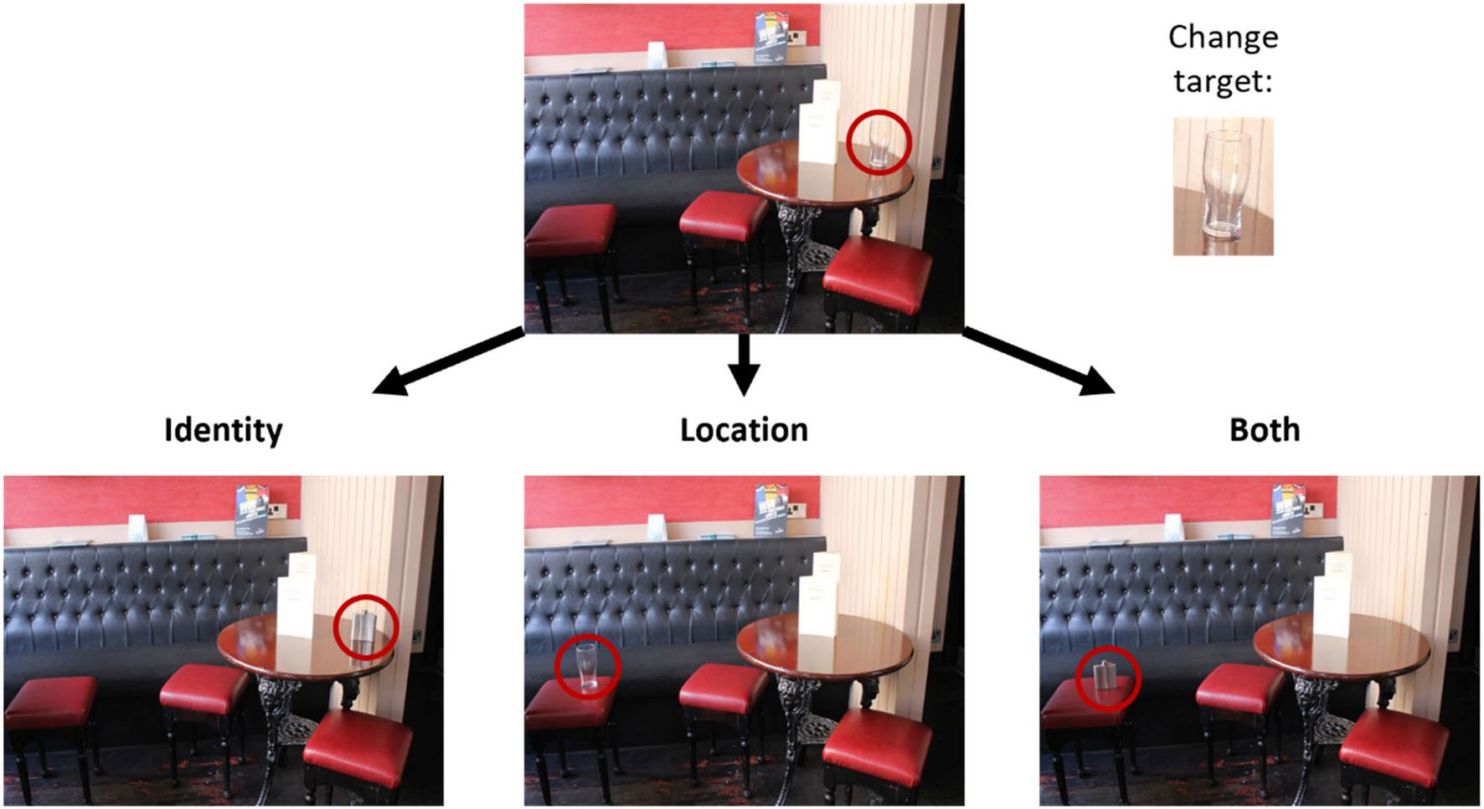
An example of the types of change implemented in our study. The original image presented during the study phase is shown at the top. During the recognition phase, the image could present a change in Location (the object moves from left to right, or vice-versa) Identity (the object becomes another object, consistent or inconsistent with the scene), and Both (the object moves and becomes another object). The critical object is shown here surrounded by a red circle, which was not present in the experiment.

We designed a VWM change detection task, in which participants were asked to detect whether a change occurred (or not) on a critical object in the scene. Some examples illustrating the type of scenes used in the study are provided in Fig. 1. Three *types of change* were implemented: (a) Location, the target object moved from left to right (or vice-versa) in the scene, (b) Identity, the object stayed in the same location, but it became another object which was either consistent or inconsistent with the scene (e.g., a *beer glass* or a *hipflask* in a restaurant), or (c) Both, the object became another object and moved in the scene (please refer to Fig. 2 for an example of the changes implemented). We fully counterbalanced the type of change (e.g., left–right or consistent-inconsistent) between trials to prevent participants from developing strategies throughout the experiment. The experiment was implemented on Experiment Builder (SR Research, 2004).

### Apparatus

Scenes were presented on a 19″ CRT Monitor IIYAMA Vision Master Pro 454 with a refresh rate of 75 Hz at a viewing distance of 60 cm, taking up a 35.81° × 26.86° (width × height) field of view. We co-registered eye movements and electrophysiological (EEG) responses. The eye-movement data was recorded using an SR Research EyeLink 1000 Desktop-based system (1000 Hz sampling rate). We performed a parallax test to define the eye dominance of each participant, and only the dominant eye was tracked. A chin rest was used to keep participants’ head position stable. EEG data were recorded using a BioSemi ActiveTwo AD box (512 Hz sampling rate) on 64 EEG channels, 4 EOG (left and right horizontal cantus, and above/below the right eye), referenced to the common mode sense (CMS; active electrode) and grounded to a passive electrode. In this study, we focused our analysis on the manual and the eye-movement responses collected during the recognition phase, whereby the EEG data will not be discussed any further.

### Materials

The stimuli consisted of 192 photographic images of indoor scenarios (e.g., bedrooms, bathrooms, etc.), of which 96 experimental (change items) and 96 fillers (no-change items), which had been used in a previous experiment by our team^78^. Each scene contained a critical object, which was either consistent or inconsistent with the context of the scene. Eight naïve participants, who were not involved in any other aspect of the study, assessed the consistency of the object in a pre-test. Each participant was presented with a subset of the photographs, as object congruency and its location within the scene were counterbalanced across four different lists. Participants were required to name the target object (cropped and presented in a separate box next to the scene) and rate the likelihood of finding the target object within the scene using a Likert scale (1–6). We obtained a mean naming agreement of 96%, and consistent objects were judged as significantly more likely (5.78 ± 0.564) than inconsistent objects (1.88 ± 1.107) using independent samples Kruskal–Wallis H test [χ^2^(1) = 616.09, *p* < 0.001]. A LMER analysis revealed that change detection accuracy for scenes which contained an inconsistent object (mean accuracy = 79.57%, SD = 8.1%) did not significantly differ from scenes containing a consistent object (mean accuracy = 76.55%, SD = 10.98%). To further ensure that the consistency of the critical object did not affect results, we repeated all analyses reported below but including trials that only had consistent objects (see Supplementary Information for the full output of these analyses), and the results largely corroborated what was found when including all items (see “Results” section). Therefore, and since the focus of the present study was to investigate the effects of location and identity changes in VWM, the consistency manipulation will not be further discussed. Readers interested in the effect of object consistency on the allocation of overt attention in the younger group and during the study phase are referred to Coco, Nuthmann and Dimigen^79^. Finally, paired *t* tests showed that the low-level visual saliency of the critical object (peak value), as computed using the classic model by Itti, Koch and Neibur^69^, did not differ significantly when the critical object was consistent or inconsistent [mean difference = − 0.003, t(95) = − 0.2, p = 0.8], nor when it was placed to the left or the right of the scene [mean difference = − 0.03, t(95) = − 1.43, p = 0.2].

### Procedure

**Figure 3:**
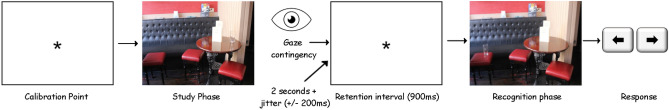
Schematic representation of a trial. Each trial starts with a drift correction. Then the study scene appears. When the gaze of the participant enters the critical object and dwells in it for 150 ms, the image stays on screen for an additional 2 s (with a jitter of 200 ms), after which a retention interval screen (a fixation cross presented for 900 ms) appears. Then, the scene appears again (recognition), and the participant has to state whether there was a change or not in the scene by pressing the keyboard. Our analysis of the eye-movements focused on responses collected during the recognition phase.

Each session started with a nine-point calibration of the eye-tracker, which was repeated any time the fixation of the participant was off by 0.5° and 1° of visual angle (horizontal and vertical) to the drift correction point (presented between trials). Each trial started with the presentation of a scene that the participant was asked to study. In this phase, a gaze contingency mechanism was used to control the presentation of the scene, and to ensure that the target object was looked at. In particular, the scene disappeared 2 s (± a jitter of 200 ms drawn from a uniform distribution) after the participant had fixated on the critical object for 150 ms. This time was added before the retention interval (a fixation cross placed in the centre of the screen for 900 ms) to prevent participants from systematically associating the last fixated object with the object that may (or not) change. If the participant did not fixate on the target object within 10 s. from the onset of the study scene, the retention interval was triggered, nevertheless. After the retention interval, the same scene was presented again (recognition phase); in half of the trials, no change occurred, whereas in the remaining trials the scene underwent one of the three changes described above. During the recognition phase, participants had 10 s to log whether they detected a change by pressing the arrow buttons on the keyboard (< no-change; > change). Such press would trigger the presentation of the next trial. If the participant exceeded the time limit of 10 s, a null response was recorded, and the next trial began. A schematic representation of the procedure is shown in Fig. 3. Each participant completed 4 practice trials followed by 96 change trials and 96 no-change trials presented in random order. A Latin Square Rotation was used to counterbalance the experimental conditions and to distribute them across 12 different lists. The task was explained using written instructions and took between 20 and 40 min to complete.

### Data analysis

#### Data processing

Analyses focused on the change trials, in which we implemented our experimental manipulations. Of the 4800 change trials (i.e., 50 participants × 96 scenes), we excluded 34 trials (0.7%) that timed out (i.e., no manual response), 50 trials (1.05%) that had a response time slower than 99% of all trials, as computed independently for each participant, and 341 further trials for scenes that were recognized at (or below) chance level. The number of change trials contributing to the analysis of manual responses was 2285 trials for the younger adults, with a by-participant average of 87.88 ± 0.65, and 2090 for the older adults, with a by-participant average of 87.06 ± 2.68. When grouping the trials contributing to the analysis of the manual responses by the type of change, we observe a by-participant average of 28.84 ± 1.56 Identity trials, 29.5 ± 1.65 Location trials, and 29.53 ± 1.9 Both trials for the younger group; 28.41 ± 2.91 Identity trials, 29.54 ± 1.79 Location and 29.12 ± 1.82 Both trials for the older adults.

Fixations and saccades were extracted from the raw gaze data using the Data Viewer software (SR Research), which performs saccade detection based on velocity and acceleration thresholds of 30° s^−1^ and 9500° s^−1^ respectively. Our analyses of the eye-movement responses focus on data collected during the recognition phase; for the analyses of this data, out of the 4375 trials considered for the analysis of manual responses, we had to exclude a further 1047 trials (23.93%) which had no fixations on the critical object during the study phase, to make sure that the object was indeed fixated and 619 trials (22.84%) where participants did not correctly detect the change during the recognition phase. The number of change trials contributing to the analysis of the eye-movement responses was a total of 1426 trials for the younger adults, with a by-participant average of 54.84 ± 8.88 trials, and a total of 1283 trials for the older adults, with a by-participant average of 53.45 ± 8.01 trials. When grouping the trials contributing to the analysis of the eye-movement responses by the type of change, we observe a by-participant average of 17.3 ± 3.80 Identity trials, 17.8 ± 4.17 Location trials, and 19.73 ± 3.7 Both trials for the younger group, and 16.87 ± 4.39 Identity trials, 18.25 ± 4.39 Location and 18.2 ± 4.05 Both trials for the older adults.

#### Dependent measures

**Figure 4:**
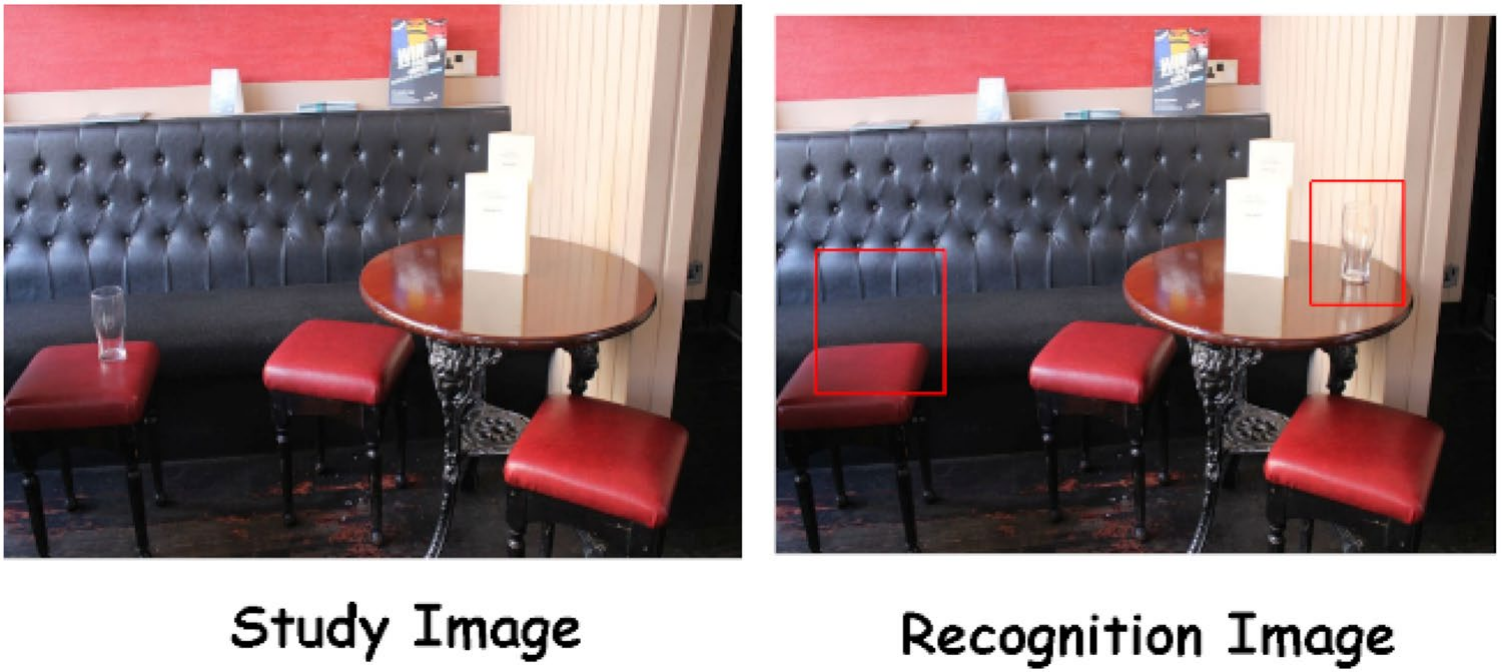
Example of the areas of interest (AOI) created for the Both and Location conditions. The AOI corresponding to the original location where the target was placed during the Study phase (Past Location) is represented by the red square on the left; the AOI corresponding to the new target location during the Recognition Phase (Current Location) is represented by the red square on the right.

We assessed change detection performance by looking at (a) *response accuracy* (a binomial variable with values of 1 for correct and 0 for incorrect response) and, on correct trials only, (b) *response time*, which was calculated from the onset of the recognition scene until the participant pressed the keyboard. Measures of eye-movement behaviour were computed using the data collected during the recognition phase. For the conditions in which the object changed in location (Location and Both), we consider two possible areas of interest that the participants could look at to support change detection: where the critical object was displayed in the scene during the recognition phase (Current Location), and where it had been displayed during the study phase (Past Location). Since in the Identity condition the object always occupied the same spatial location within the scene (i.e., the Past Location was the same as the Current Location), in this condition we only considered one area of interest (please refer to Fig. 4 for a visualization of the areas of interests). We computed three different eye-movement measures: (a) the *incoming saccade amplitude* towards the areas of interest, which reflects the area of the peripheral visual field from which participants were able to select the target to fixate^15^, (b) the *latency to the first fixation*, which is the time between the onset of the array and the first fixation on the critical object and indicates the time taken to identify the area of interest (see^80^ for an example in visual search); and *first-pass gaze duration*, that is the summed duration of all fixations during the first inspection of the area of interests, and points at the effort to retrieve information about the occurred change^81^. Response times, latencies to the first fixation, and first-pass duration were all z-scored independently by age group to account for the general slowing effect associated with aging^50^.

#### Statistical modelling

We use generalized and linear-mixed effects modelling (G/LMER) as implemented in the lme4 R package^82^ to provide statistical inference. This approach makes it possible to directly tackle the intrinsic variability of participants and scenes on the dependent measures^83^.

The fixed effects considered in the models, and centred to reduce collinearity, are *Change Type* (Location, Both, and Identity, which was also the reference level) and the between-participant *Group* variable (Younger = − 0.5 and Older = 0.5). For the eye-movement analysis focusing on changes to object location, we only consider two out of the three factors of the Change Type variable (i.e., Location = − 0.5 and Both = 0.5) and include an additional predictor to compare the role played by *Object Location* (Current Location = − 0.5, and Past Location = 0.5). We considered both main effects and interactions for each predictor (i.e., a full fixed-effect structure). The random effects are *Participant* (50) and *Scene* (89) introduced as intercepts. Additionally, we evaluated whether the low-level visual saliency of the critical object (peak value), as computed using the classic model by Itti, Koch and Neibur^69^, influenced the eye movement measures on the critical object in systematically different ways between the two age groups. To do so, LMER models were built to predict each eye-movement measure as a function of Saliency, Group, and Type of Change.

In the table of results, we reported the beta coefficients, t-values (LMER), z-values (GLMER), and p-values of all predictors, and highlighted the significant results in bold. The level of significance was calculated from an *F* test based on the Satterthwaite approximation to the effective degrees of freedom^84^, whereas p-values in GLMERs were based on asymptotic Wald tests.

## Results

**Figure 5:**
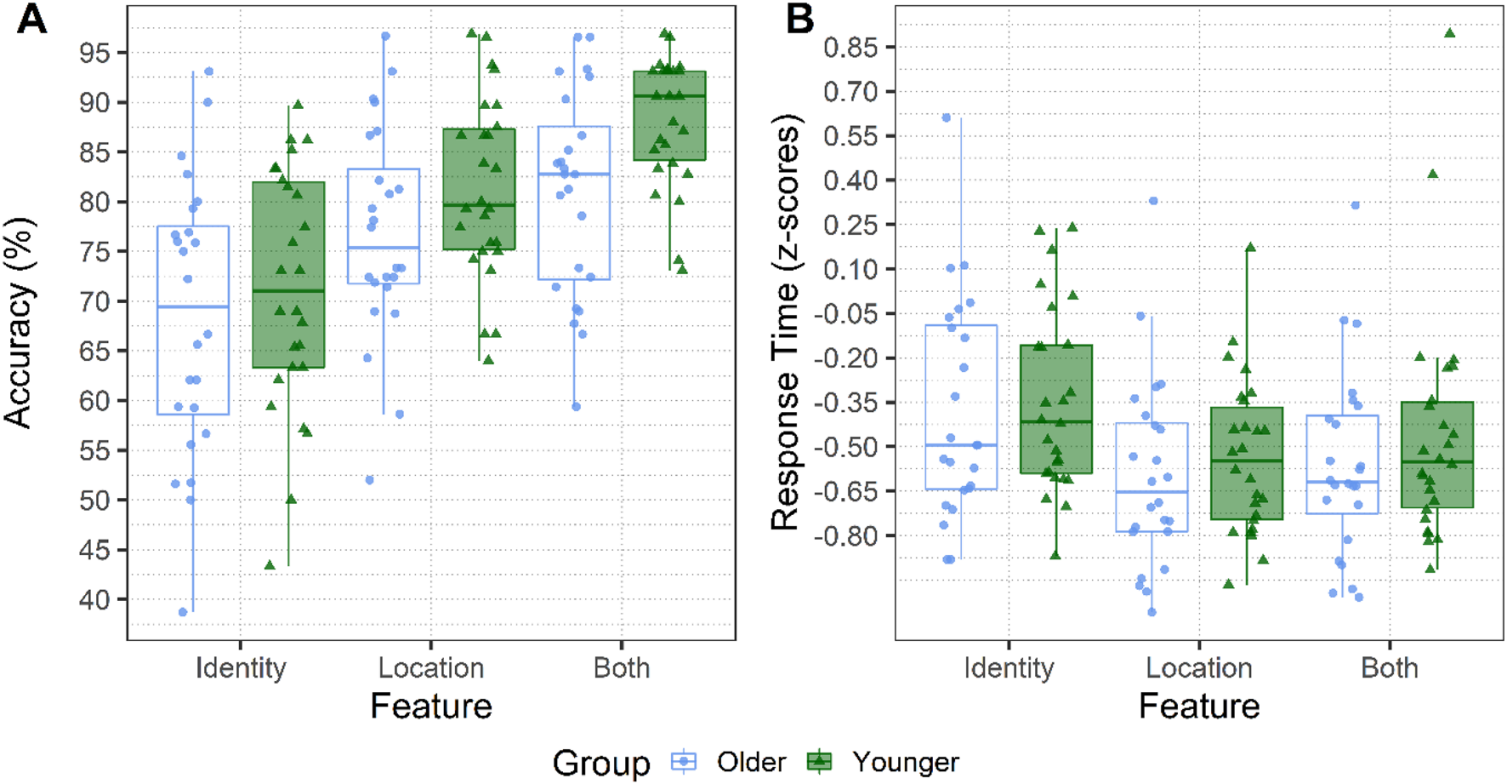
Manual responses: (**A**) The percentage accuracy of the change detection task. (**B**) The response time for correct trials is z-scored. Both measures are plotted on the y-axis as a function of the different Types of Change (Identity, Location, and Both). The two Groups of participants are compared within each panel. The Younger group is depicted in green, whereas the Older group is depicted in blue. The hinges of the boxplots represent the 25th and 75th percentiles of the measure (lower and upper quartiles). The horizontal line represents instead the median of the distribution. Each dot indicates the by-participant average for that factor.

Figure 5A shows the percentage of accurately recognized changes as a function of their type comparing the younger and older group. Figure 5B displays the response time (in z-scores) taken to provide an accurate choice; the reader is referred to Table 2 for the model coefficients. For accuracy, results revealed a significant main effect of Group, whereby older adults were overall less accurate than younger adults. In addition, we observed a significant main effect of Type of Change: accuracy was significantly higher when the critical object changed in Both features than when it only changed in Identity, and this was especially the case for younger adults (as indicated by the significant interaction with Group). When looking at the normalized response times, we corroborate that correctly detecting a change in Identity is more effortful, and so it requires a longer time (main effect of Both and Location); groups did not significantly differ.

**﻿Table 2:** Generalized and linear mixed effects model output for the manual responses of recognition accuracy (correct vs. incorrect trials) and response time (correct trials only).

Dependent variable	Predictor	β	SE	z-value/t-value	Pr (> |z|)
Response Accuracy	Intercept	1.52	0.11	13.18	**< 0.001**
	Group	0.36	0.15	2.27	**0.02**
	Both vs. Identity	0.97	0.11	8.15	**< 0.001**
	Location vs. Identity	0.1	0.11	0.95	0.3
	Group × Both vs. Identity	0.49	0.23	2.08	**0.04**
	Group × Location vs. Identity	− 0.08	0.22	− 0.37	0.7
Response Time (z-score)	Intercept	− 0.46	0.04	− 9.91	**< 0.001**
	Group	0.06	0.08	0.7	0.48
	Both vs. Identity	− 0.07	0.03	− 2.26	**0.02**
	Location vs. Identity	− 0.16	0.03	− 5.06	**< 0.001**
	Group × Both vs. Identity	0.08	0.06	1.28	0.2
	Group × Location vs. Identity	0.01	0.06	0.14	0.9

Predictors centred and standardized entered in the G(L)MER were: Group (Older = − 0.5 and Younger = 0.5) and Type of Change (Location, Both—Identity as reference level). We report the *β*, the standard error, the *t*-value, and the *p*-value. The random effects introduced as intercepts were Participants (50) and the unique identifier of Scene item (89).

**Figure 6:**
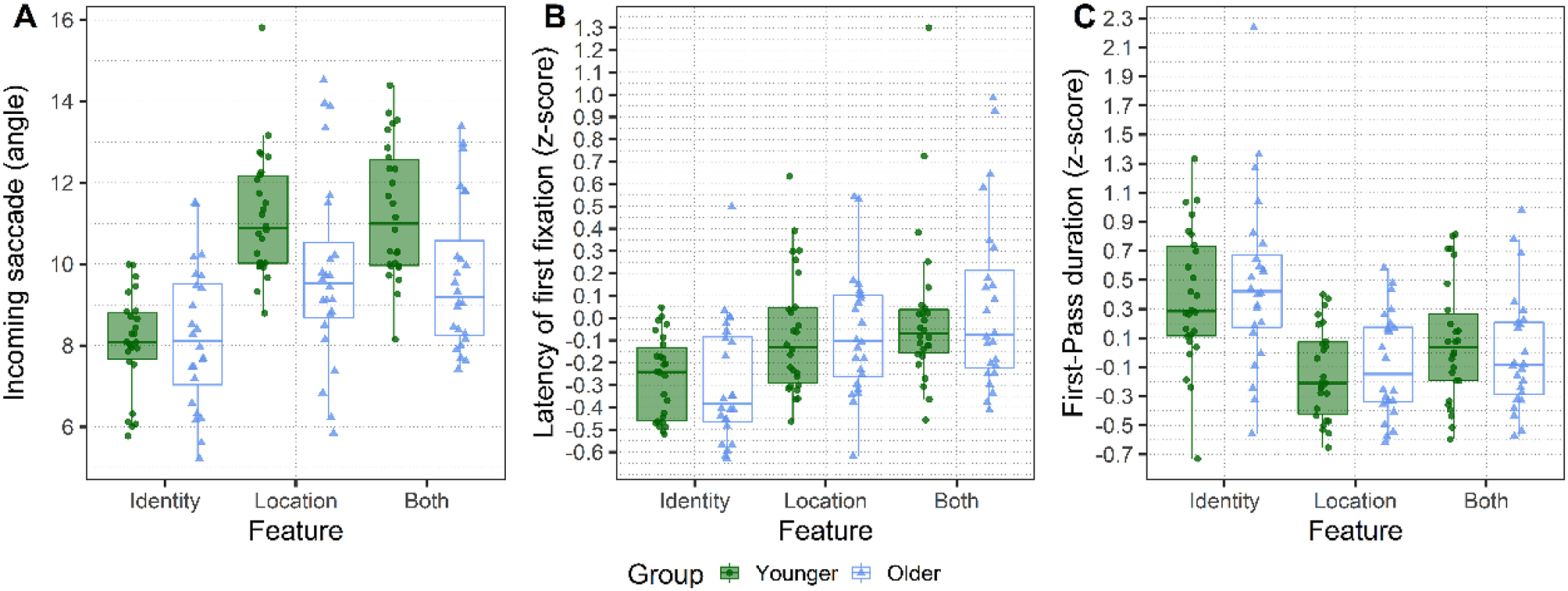
Eye-movement measures on the Current Location: (**A**) Incoming saccade amplitude of first fixation on the critical object in degrees of visual angle, (**B**) Latency of the first fixation on the critical object (z-scores), and (**C**) the summed duration of all fixations on the critical object before exiting it for the first time (First-Pass, also in z-scores). The two Groups of participants are compared within each panel. The Younger group is depicted in green, whereas the Older group is depicted in blue. The hinges of the boxplots represent the 25th and 75th percentiles of the measure (lower and upper quartiles). The horizontal line represents instead the median of the distribution. Each dot indicates the by-participant average for that factor.

When considering eye-movement measures directed to the Current Location (see Fig. 6 for visualization and refer to Table 3 for the model coefficients), results revealed a significant main effect of Type of Change on all eye movement measures considered. Contrasts revealed that incoming saccades were longer in the conditions that implied a relocation of the object (Location and Both) compared to when the object only changed in Identity. We also found a significant main effect of Group on saccade amplitude, as older participants made shorter incoming saccades than younger participants, and a significant interaction indicating that this difference was driven by the Both condition. Targets were fixated later (as shown by the longer latencies to the first fixation) and for less time (as indexed by the shorter first-pass gaze durations) when Both features were changed compared to when only the Identity was changed. First-pass gaze durations were also shorter in the Location than in the Identity condition.

**﻿Table 3:** Linear mixed effects model output for the eye-tracking measures on the Current Location during recognition (correct trials only): incoming saccade amplitude, latency to first fixation (z-score) and first-pass gaze duration (z-score) on the critical object.

Dependent variable	Predictor	β	SE	t-value	Pr (> |z|)
Incoming saccade amplitude	Intercept	9.56	0.2	47.28	**< 0.001**
	Group	− 0.85	0.31	− 2.68	**0.01**
	Both vs. Identity	1.6	0.36	4.39	**< 0.001**
	Location vs. Identity	1.6	0.36	4.35	**< 0.001**
	Group × Both vs. Identity	− 1.68	0.73	− 2.28	**0.02**
	Group: × Location vs. Identity	− 0.62	0.74	− 0.87	0.4
Latency of First Fixation (z score)	Intercept	− 0.07	0.04	− 1.70	0.09
	Group	0.01	0.06	0.19	0.85
	Both vs. Identity	0.3	0.05	6.11	**< 0.001**
	Location vs. Identity	0.09	0.05	1.86	0.06
	Group × Both vs. Identity	0.07	0.1	0.73	0.46
	Group × Location vs. Identity	0.001	0.1	0.02	0.99
First-Pass Duration (z score)	Intercept	0.15	0.05	2.92	**0.005**
	Group	0.02	0.09	0.19	0.85
	Both vs. Identity	− 0.15	0.06	− 2.4	**0.02**
	Location vs. Identity	− 0.5	0.06	− 7.78	**< 0.001**
	Group × Both vs. Identity	− 0.13	0.13	− 1.02	0.31
	Group × Location vs. Identity	0.02	0.13	0.13	0.89

Predictors centred and standardized entered were Group (Younger = − 0.5 and Older = 0.5) and Type of Change (Location, Both—Identity as reference level). We report the *β*, the standard error, the *t*-value and the *p*-value. The random effects introduced as intercept were Participants (50) and the unique identifier of Scene item (89).

**Figure 7:**
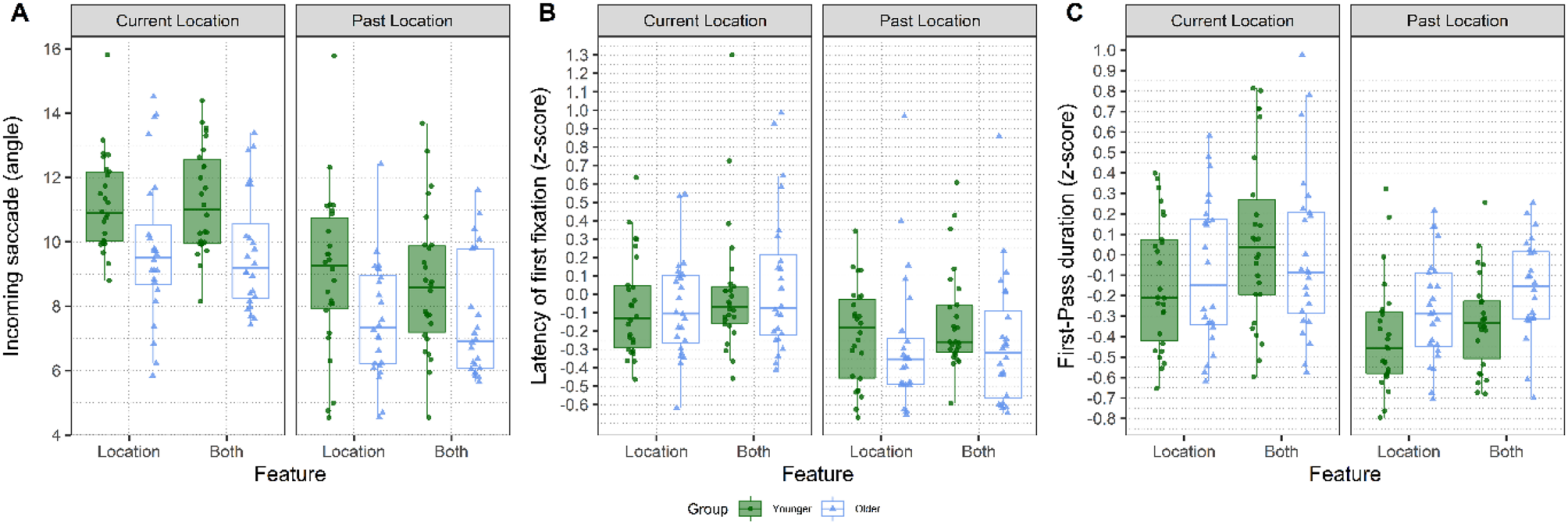
Eye-movement measures comparing Current and Past Location: Here, we focus our comparison between the two conditions that involved a change in location of the target object (Location, Both). Current Location is where the object is now displayed in the recognition phase. Past location instead is where the target object was during the study phase. (**A)** Incoming saccade amplitude of the first fixation to the critical object in degrees of visual angle, (**B**) Latency of the first fixation on the critical object (z-scores), and (**C**) the sum of all fixations on the critical object before exiting it for the first time (First-Pass, also in z-scores). The Current Location and the Past Location are compared side-by-side and two Groups of participants within each panel. The Younger group is depicted in green, whereas the Older group is depicted in blue. The hinges of the boxplots represent the 25th and 75th percentiles of the measure (lower and upper quartiles). The horizontal line represents instead the median of the distribution. Each dot indicates the by-participant average for that factor.

We further analysed only the conditions which involved a spatial relocation of the critical object in the scene (i.e., Location and Both) and compared eye-movement measures to the Current Location of the object (i.e., the results just presented) with those associated with the Past Location where the critical object was positioned during the study phase (see Fig. 7 for visualization, and Table 4 for the model coefficients). These analyses confirmed shorter saccades in the older compared to the younger adults, and they additionally showed that both groups made shorter saccades to the Past Location compared to the Current Location. For the latency of the first fixation, we found a significant main effect of object Position and a significant main effect of Type of Change: first fixations were faster when directed to the Past compared to the Current Location, and when the change involved only the Location compared to Both features of the object. Moreover, the first-pass gaze duration was significantly longer when the object changed in Both features than when it only changed in Location. Once the object was fixated, first-passes were longer onto the Current Location compared to the Past location. There was also a significant interaction between Group and Object Position whereby older adults displayed relatively longer first-pass at the Past Location compared to the Current Location than younger adults.

**﻿Table 4:** Linear mixed effects model output for the eye-tracking measures during recognition (correct trials only) on the Current Location and Past Location, focusing on the conditions in which the critical object changed position (Both and Location).

Dependent variable	Predictor	β	SE	t-value	Pr (> |z|)
Incoming saccade amplitude	Intercept	9.33	0.2	46.14	**< 0.001**
	Group	− 1.37	0.29	− 4.69	**< 0.001**
	Change Type	0.09	0.24	0.38	0.71
	Position	− 2.39	0.25	− 9.73	**< 0.001**
	Group × Change Type	− 0.35	0.48	− 0.72	0.5
	Group × Position	0.52	0.48	1.09	0.28
	Change Type × Position	0.25	0.48	0.52	0.6
	Group × Change Type × Position	0.31	0.96	0.33	0.74
Latency of First Fixation (z-score)	Intercept	− 0.09	0.04	− 2.05	**0.04**
	Group	− 0.01	0.07	− 0.22	0.82
	Change Type	0.09	0.03	2.51	**0.01**
	Position	− 0.26	0.03	− 7.32	**< 0.001**
	Group × Change Type	− 0.05	0.07	− 0.67	0.5
	Group × Position	− 0.09	0.07	− 1.3	0.19
	Change Type × Position	− 0.05	0.07	− 0.75	0.45
	Group × Change Type × Position	− 0.17	0.14	− 1.24	0.22
First-Pass Duration (z-score)	Intercept	− 0.15	0.04	− 4.13	**< 0.001**
	Group	0.07	0.06	1.14	0.26
	Change Type	0.12	0.03	3.42	**< 0.001**
	Position	− 0.3	0.04	− 8.33	**< 0.001**
	Group × Change Type	− 0.001	0.07	− 0.02	0.99
	Group × Position	0.19	0.07	2.67	**0.008**
	Change Type × Position	− 0.07	0.07	− 1.03	0.3
	Group × Change Type × Position	0.1	0.14	0.72	0.47

Predictors centred and standardized entered were: Group (Younger = − 0.5 and Older = 0.5) Change Type (Location = − 0.5 and Both = 0.5), Position (Current Location = − 0.5 and Past Location = 0.5). We report the *β*, the standard error, the *t*-value and the *p*-value. The random effects introduced as intercept were Participants (50) and the unique identifier of Scene item (89).

Finally, the peak salience of the critical object had a significant effect on the latency of the first fixation, as more salient objects were looked at faster than less salient ones (Table 5). However, salience did not significantly predict first-pass gaze durations nor incoming saccade amplitudes, and we did not find any significant interaction between this variable and either Group or Type of Change.

**﻿Table 5:** Linear mixed effects model output for the effects of saliency on eye-tracking measures on the Current Location during recognition (correct trials only): incoming saccade amplitude, latency to first fixation (z-score) and first-pass gaze duration (z-score) on the critical object.

Dependent variable	Predictor	β	SE	t-value	Pr (> |z|)
Incoming saccade amplitude	Intercept	10.45	0.43	24.07	**< 0.001**
	Saliency	− 1.01	0.59	− 1.71	0.09
	Group	− 1.39	0.68	− 2.04	**0.04**
	Both versus Identity	− 0.32	0.96	− 0.33	0.74
	Location versus Identity	2.09	0.97	2.16	**0.03**
	Group × Saliency	0.89	0.93	0.96	0.34
	Saliency × Both vs Identity	2.50	1.37	1.82	0.07
	Saliency × Location vs Identity	− 1.32	1.38	− 0.95	0.34
Latency of First Fixation (z score)	Intercept	0.33	0.08	3.96	**< 0.001**
	Saliency	− 0.33	0.10	− 3.12	**0.002**
	Group	0.03	0.12	0.22	0.82
	Both versus Identity	0.43	0.15	2.86	**0.004**
	Location versus Identity	0.4	0.15	2.61	**0.009**
	Group × Saliency	0.0009	0.15	0.006	0.99
	Saliency × Both vs Identity	− 0.28	0.22	− 1.3	0.19
	Saliency × Location vs Identity	− 0.36	0.22	− 1.67	0.09
First-Pass Duration (z score)	Intercept	− 0.003	0.08	− 0.05	0.96
	Saliency	0.09	0.10	0.92	0.36
	Group	− 0.02	0.14	− 0.14	0.89
	Both versus Identity	0.06	0.17	0.34	0.73
	Location versus Identity	− 0.47	0.17	− 2.70	**0.007**
	Group × Saliency	− 0.003	0.17	− 0.02	0.98
	Saliency × Both vs Identity	− 0.24	0.24	− 0.99	0.32
	Saliency × Location vs Identity	0.08	0.24	0.32	0.75

Predictors centred and standardized entered were Saliency, Group (Younger = − 0.5 and Older = 0.5) and Type of Change (Location, Both—Identity as reference level). We report the *β*, the standard error, the *t*-value and the *p*-value. The random effects introduced as intercept were Participants (50) and the unique identifier of Scene item (89).

## Discussion

There currently is no consensus as to whether aging selectively affects the ability to form and successfully maintain temporary bindings about the identity and location of objects in short-term visual working memory. Age-related declines in the successful formation of temporary bindings of object features in visual short-term memory have at times been observed in studies that have used arrays of decontextualised objects^12,37^. Our study aimed to determine whether VWM representations of individual features or their combination are impaired by healthy ageing when the task involves a naturalistic rather than an artificial context. Younger and older adults were asked to detect changes to the identity of an object, its location, or both features, in naturalistic photographs. We then compared their recognition performance as well as their eye movements to investigate the interaction between VWM and overt visual attention.

Results revealed that older adults were overall less accurate than younger adults, which confirms previous findings from studies that used simple arrays of visual objects^15,85^ as well as from studies using more naturalistic stimuli^48,49^. An overall drop in change detection performance due to age was expected, given that reductions in processing speed and cognitive functioning are known to accompany healthy aging^4,50,51^. Costello and colleagues^49^, for example, observed that, when change detection performance was considered by itself, older adults performed significantly worse than younger adults; however, this age-related effect did not hold when the perceptual speed of participants was included in the statistical model and accounted for over 70% of the variance when considered alone. Our main focus, however, was on determining whether specific mechanisms are impaired more than others rather than assessing overall age-related reductions in change detection abilities. In particular, we investigated whether the ability to maintain in VWM individual features versus feature combinations would show signs of selective disruption in older compared to younger adults. Our results showed similarities in both age groups. Changes that involved both the identity and the location of an object had the highest likelihood to be detected, and this effect was more pronounced for younger adults. For both groups, accurate detections were faster when the critical object changed in location compared to when it only changed in identity, which instead led to the slowest detections. The first implication of these results is that maintaining VWM representations of feature conjunctions in naturalistic contexts does not impose additional costs compared to isolated features, which contrasts some of the results obtained with object arrays^8,9,86,87^. Second, although there was an overall reduction in the detection performance due to age, both groups were similarly affected by the type of change, indicating that older adults were not disproportionately impaired in maintaining VWM representations of objects’ location^33,34^, their identity^41^ or the association between these features^38^.

Evidence showing that maintaining feature conjunctions in VWM is costly mainly comes from studies that used arrays of artificial objects^13,14,16,18,37^ (but see, e.g.^18–20^ for contrasting evidence showing preserved binding abilities in aging). The advantage of using artificial displays is that they allow great control of low- and high-level stimulus properties, and certainly, this approach has been necessary to uncover the basic mechanisms of VWM. However, insights from these studies cannot be easily generalised to more naturalistic scenarios such as complex photographic scenes, which are inherently different from artificial object arrays. Research on scene perception has convincingly shown that contextual information can facilitate VWM performance^88–91^. Observers can rapidly extract low- and high-level information from the display and learn the statistical regularities of the scene to efficiently integrate the spatial, semantic, and functional relationships between objects^92^, which enables predictions about which objects are likely to be found and where^91,93,94^. Thus, it is plausible to assume that participants in our task used contextual information to encode more effectively the identity and location of objects, which may explain the superior performance in the Both condition, and relatedly the faster detection of location compared to identity changes. Detections of location changes can occur through memory for the scene layout, whereas identity changes require access to semantic knowledge about the object. Moreover, the fact that changes to feature conjunctions (i.e., identity and location) were better detected than changes that involved a single feature, such as the object identity only, favours a probabilistic^95^ and dynamically adaptable VWM capacity account^9,86,87^, where objects are conceptualized as hierarchical bundles of features^60^ rather than bounded units^96^.

The inclusion of eye movement responses in our study helped to uncover the attentional strategies employed by younger and older adults to successfully recognize changes. Relative to the target (i.e., Current Location) first, the only difference between the two groups was found in the amplitude of the saccade. Saccades were larger for younger than older participants, reflecting age-related reductions in the useful field of view (in line with, e.g.^15,66^). Furthermore, this age-related difference in saccade amplitude was especially pronounced when the object changed in both identity and location, which links to the significant interaction observed in detection accuracy, whereby younger adults showed a greater memory advantage compared to older adults when the object changed in both location and identity. The fact that older adults’ performance did not benefit as much from a change in both features could qualitatively indicate that they have reduced access to semantic information about the object, i.e., its identity^35,37,38^.

All other significant differences observed in the eye movements to the Current Location were only associated with the type of change, i.e., no significant interaction with the Group variable, further indicating that, regardless of age, overt attention was similarly allocated by both groups to successfully detect changes. When looking at the latency of the first fixation to the critical object, we found slower latencies when the object changed in both identity and location compared to a change only in identity. This processing cost on the latency may be due to the conjunctive nature of the change (i.e., two features instead of one), which corroborates the increased response times observed in search tasks (e.g.^67^). The duration of this first fixation was significantly longer when the critical object only changed in identity compared to when the change involved its relocation. Since fixation durations reflect the time needed to acquire sufficient information about a stimulus^97^, this finding complements our results on detection accuracy, which was the lowest in the identity condition. This result also confirms that retrieving semantic information about the identity of the object to drive the detection requires more extensive processing than retrieving its spatial information relative to the scene context. The integration of object-to-scene information is known to be costly as the competition between the identity of the object and the semantic context of the scene needs to be resolved, and this is reflected in longer fixations^98–101^. Moreover, this result lends additional support to frameworks of VWM postulating independent encoding of features (e.g.^60^) as detecting a change in feature conjunctions requires access to only one of the two features, hence the reduced attentional demands.

We also focused on the two change conditions involving relocation of the object (Location, Both) to better detail the link between overt attention and VWM when semantic processing is also involved. To do so, we compared eye-movement measures associated with the location where the critical object was in the study phase (Past Location) to those associated with where it instead moved during the recognition phase (Current Location). The amplitude of the saccade was shorter in older than younger adults, which confirmed the results observed when only the Current Location was considered. We also confirmed that participants took significantly longer to look at the critical object when it changed also in identity compared to a change only in location. These results also showed that, when the critical object shifted to a new location in the scene, the previously occupied location rapidly attracted gaze, as reflected in shorter latencies and saccades to the Past compared to the Current location. Possibly, participants made use of a VWM template of the scene to covertly acquire location information, and this extra-foveal processing guided overt eye movements towards the now empty location. This qualitative interpretation is in line with the notion that when spatial locations are efficiently stored in VWM tasks, the deployment of overt attention may be facilitated^68^. It should be noted that in our current design when the critical object moved to a new location in the recognition phase it left its original location empty. This may have been perceived as a change in the spatial configuration of the scene^6,94^, which in turn may have facilitated detection in these conditions. We are addressing this limitation in current research, in which location changes involve the swap in the position of two objects (the critical object and a swap object) to maintain the overall spatial configuration unchanged. This should enable us to better clarify the relative contributions of memory for the identity of an object versus memory for its location.

Interestingly, we also observed significantly longer first-passes on the Current location compared to the Past location, especially in younger compared to older adults. These results are consistent with a recent study by Wynn and colleagues^55^ which investigated the ability of younger and older adults to detect changes to the position of abstract objects displayed at various locations. After the initial study phase, which was followed by a retention period during which the screen was left blank, participants were shown another display where objects either occupied the same locations on the screen or not. The key observation of this study was that in the retention interval, older adults had a greater tendency to reinstate fixations made to the locations where objects had been encoded, which indicates a compensatory strategy to support the maintenance of the spatial layout of the scene. In line with Wynn and colleagues, we argue that our result of comparatively longer looking times on the Past location observed in the older participants suggests that they had to accumulate more information from the memory location to correctly identify the change, potentially reflecting a compensatory attentional strategy in this group to support a possible deficit in accessing VWM representations.

Finally, we examined the effects of low-level visual saliency on the eye-movement responses to the critical object and found that the time to the first fixation was impacted (i.e., faster for more salient objects), which is in line with previous findings^69–72^. However, saliency did not significantly affect the other eye movement metrics considered, and importantly, it did not interact with Group. Coupled with the fact that the natural saliency of the critical object balanced out across all scenes, this result suggests that eye movements were mainly driven by high-level features and top-down VWM processes, which corroborates what was found elsewhere^74–77^.

As a point of potential caution, we found significant differences between younger and older adults in their neuropsychological profiles (see Table 1) which could have affected the results of the present study. Post-hoc correlation analyses however revealed that there was no significant correlation between the scores achieved on the neuropsychological tests and accuracy or response times to the change detection task. This suggests that the expected reductions in cognitive abilities typically found in healthy older adults^4,50,51^ did not seem to bear any significant consequence on the results presented in the current study.

We believe that the present study contributes to the still limited research on cognitive processes situated in naturalistic scenarios, which is revealing important differences that can no longer be neglected. For example, eye movements substantially differ when exploring still frames of scenes, or videos, compared to the real world^102^, and encoding of information in real-life scenarios results in superior memory performance compared to when the same information is encoded in a laboratory environment^103^. Although photographs of real-life scenes, such as those used here, are more meaningful and ecologically valid than simple shapes, they still do not capture other spatial and temporal aspects emerging when our cognitive interaction takes place in the real world. Therefore, we advocate for research that is increasingly more engaged with ecologically valid methods to investigate attention allocation during real-life—a transition that is nowadays possible thanks to the availability of portable and affordable eye-tracking devices.

## Supplementary Information

Supplementary Tables.

## Data Availability

The data and R script to analyse the results of this manuscript are available on the Open Science Framework at: https://osf.io/k5fwx/?view_only=5615c15bb3f34cb5b6c72bbc56d83fdb. Miniatures of the scenes used in the study are available at: https://osf.io/sjprh/.
