# Supplementary material for: Similar mechanisms of temporary bindings for identity and location of objects in healthy ageing: an eye-tracking study with naturalistic scenes
Source: Sci Rep. 2022 Jul 1;12:11163. doi: 10.1038/s41598-022-13559-6 (PMC9249875; doi:10.1038/s41598-022-13559-6)
Supplement: Supplementary file 1 — Supplementary Tables. [file 41598_2022_13559_MOESM1_ESM.docx]

**Supplementary Information**

For

**Similar mechanisms of temporary bindings for identity and location of objects in healthy ageing: An eye-tracking study with naturalistic scenes**

Giorgia D’Innocenzo^1*^, Sergio Della Sala^2^ & Moreno I. Coco^1,3*^

^1^ Centro de Investigação em Ciência Psicológica (CICPSI), Faculdade de Psicologia, Universidade de Lisboa, Lisbon, Portugal

^2^ Human Cognitive Neuroscience, Department of Psychology, University of Edinburgh, Edinburgh, UK

^3^ Department of Psychology, “Sapienza”, University of Rome, Italy

^4^IRCCS Santa Lucia, Rome, Italy

*Correspondence can be addressed to Giorgia D’Innocenzo (giorgiadinnocenzo@psicologia.ulisboa.pt) or Moreno Coco (moreno.cocoi@gmail.com)

In this Supplementary Information we report the results of the re-analyses of all data but including only trials in which the changed target was consistent with the scene. Results replicated our overall findings, with only some minor differences: 1) the difference in response time in the Location versus the Identity condition (Table S1) is no longer significant; 2) the main effect of Type of Change on the latency of the first fixation on the critical object when we compared eye movements to the Present vs Past location (Table S3) is also no longer significant. These differences likely arise because the re-analyses are carried out on half of the available trials (i.e., only the consistent trials), resulting in reduced statistical power.

**Supplementary Table S1**

Table S1: Generalised and linear mixed effects model output for accuracy (correct vs. incorrect trials) and response time (correct trials only), considering only trials with consistent items

| Dependent  Variable | Predictor | β | SE | z-value/t-value | Pr ( > \| z \| ) |
| --- | --- | --- | --- | --- | --- |
| Response  Accuracy | Intercept | 1.52 | 0.14 | 11.02 | **<0.0001** |
|  | Group | 0.49 | 0.19 | 2.52 | **0.01** |
|  | Both vs. Identity | 1.19 | 0.20 | 5.91 | **<0.0001** |
|  | Location vs. Identity | -0.24 | 0.22 | -1.14 | 0.25 |
|  | Group x Both vs. Identity | 0.8 | 0.34 | 2.32 | **0.02** |
|  | Group x Location vs. Identity | -0.07 | 0.31 | -0.24 | 0.81 |
| Response  Time (z-score) | Intercept | -0.5 | 0.05 | -9.95 | **<0.0001** |
|  | Group | 0.08 | 0.09 | 48.19 | 0.41 |
|  | Both vs. Identity | -0.18 | 0.04 | -4.01 | **<0.0001** |
|  | Location vs. Identity | 0.02 | 0.09 | 0.21 | 0.8 |
|  | Group x Both vs. Identity | 0.08 | 0.09 | 0.88 | 0.38 |
|  | Group x Location vs. Identity | 0.02 | 0.09 | 0.21 | 0.83 |

*Note*. Predictors centred and standardized entered in the G(L)MER were: Group (Older = -.5 and Younger = .5) and Type of Change (Location, Both – Identity as reference level). We report the *β*, the standard error, the *t*-value, and the *p*-value. The random effects introduced as intercepts were Participants (50) and the unique identifier of Scene item (89).

**Supplementary Table S2**

Table S2. Linear mixed effects model output for the eye-tracking measures on the Current Location during recognition (correct trials), considering only trials with consistent items

| Dependent  Variable | Predictor | β | SE | t-value | Pr ( > \| z \| ) |
| --- | --- | --- | --- | --- | --- |
| Incoming  saccade  amplitude | Intercept | 9.34 | 0.23 | 40.98 | **<0.0001** |
|  | Group | -0.82 | 0.41 | -1.97 | 0.054 |
|  | Both vs. Identity | 2.45 | 0.51 | 4.78 | **<0.0001** |
|  | Location vs. Identity | 1.12 | 0.52 | 2.17 | **0.03** |
|  | Group x Both vs. Identity | -3.09 | 1.04 | -2.96 | **0.003** |
|  | Group: x Location vs. Identity | -0.41 | 1.05 | -0.39 | 0.69 |
| Latency of  First Fixation  (z score) | Intercept | -0.11 | 0.05 | -2.35 | **0.02** |
|  | Group | 0.03 | 0.07 | 0.48 | 0.63 |
|  | Both vs. Identity | 0.28 | 0.07 | 4.06 | **<0.0001** |
|  | Location vs. Identity | 0.25 | 0.07 | 3.60 | **0.0003** |
|  | Group x Both vs. Identity | -0.06 | 0.14 | -0.47 | 0.64 |
|  | Group x Location vs. Identity | 0.03 | 0.14 | -0.47 | 0.64 |
| First-Pass  Duration  (z score) | Intercept | 0.05 | 0.06 | 0.86 | **0.39** |
|  | Group | 0.02 | 0.10 | 0.23 | 0.82 |
|  | Both vs. Identity | -0.20 | 0.08 | -2.37 | **0.02** |
|  | Location vs. Identity | -0.36 | 0.09 | -4.24 | **<0.0001** |
|  | Group x Both vs. Identity | -0.14 | 0.17 | -0.84 | 0.40 |
|  | Group x Location vs. Identity | 0.03 | 0.17 | 0.19 | 0.85 |

*Note*. Predictors centered and standardized entered were Group (Younger = -.5 and Older = .5) and Type of Change (Location, Both – Identity as reference level). We report the *β*, the standard error, the *t*-value and the *p*-value. The random effects introduced as intercept were Participants (50) and the unique identifier of Scene item (89).

**Supplementary Table S**

Table S3. Linear mixed effects model output for the eye movement measures during recognition (correct trials) collected on two possible Object Position (Current Location, Past Location) and considering only consistent items

| Dependent  Variable | Predictor | β | SE | t-value | Pr ( > \| z \| ) |
| --- | --- | --- | --- | --- | --- |
| Incoming  saccade  amplitude | Intercept | 9.34 | 0.21 | 44.42 | **<0.0001** |
|  | Group | -1.7 | 0.35 | -4.9 | **<0.0001** |
|  | Object Position | -2.29 | 0.35 | -6.55 | **<0.0001** |
|  | Type of Change | 0.05 | 0.35 | 0.16 | 0.87 |
|  | Group x Object Position | 0.62 | 0.69 | 0.90 | 0.37 |
|  | Group x Type of Change | -0.76 | 0.70 | -1.09 | 0.27 |
|  | Object Position x Type of Change | -1.26 | 0.69 | -1.81 | 0.07 |
|  | Group x Object Position x Type of Change | 1.13 | 1.38 | 0.82 | 0.41 |
| Latency of  First Fixation  (z-score) | Intercept | -0.08 | 0.05 | -1.66 | 0.10 |
|  | Group | -0.06 | 0.08 | -0.76 | 0.45 |
|  | Object Position | -0.23 | 0.05 | -4.64 | **<0.0001** |
|  | Type of Change | -0.02 | 0.05 | -0.46 | 0.65 |
|  | Group x Object Position | -0.16 | 0.10 | -1.57 | 0.12 |
|  | Group x Type of Change | -0.07 | 0.10 | -0.68 | 0.50 |
|  | Object Position x Type of Change | -0.08 | 0.10 | -0.82 | 0.41 |
|  | Group x Object Position x Type of Change | -0.06 | 0.20 | -0.29 | 0.77 |
| First-Pass  Duration  (z-score) | Intercept | -0.18 | 0.04 | -4.87 | **<0.0001** |
|  | Group | 0.08 | 0.07 | 1.12 | 0.27 |
|  | Object Position | -0.20 | 0.05 | -3.94 | **<0.0001** |
|  | Type of Change | 0.02 | 0.05 | 0.33 | 0.74 |
|  | Group x Object Position | 0.17 | 0.10 | 1.74 | 0.08 |
|  | Group x Type of Change | 0.01 | 0.10 | 0.01 | 0.92 |
|  | Object Position x Type of Change | -0.11 | 0.10 | -1.15 | 0.25 |
|  | Group x Object Position x Type of Change | 0.15 | 0.20 | 0.77 | 0.44 |

*Note*. Predictors centred and standardized entered were: Group (Younger = -.5 and Older = .5) Change Type (Location = -.5 and Both = .5), Position (Current Location = -.5 and Past Location = .5). We report the *β*, the standard error, the *t*-value and the *p*-value. The random effects introduced as intercept were Participants (50) and the unique identifier of Scene item (89).
